# Supplementary material for: Electronegative LDL Is Associated with Plaque Vulnerability in Patients with Ischemic Stroke and Carotid Atherosclerosis
Source: Antioxidants (Basel). 2023 Feb 10;12(2):438. doi: 10.3390/antiox12020438 (PMC9952764; doi:10.3390/antiox12020438)
Supplement: Supplementary file 1 [file antioxidants-12-00438-s001.zip › antioxidants-2142155-SI.pdf]

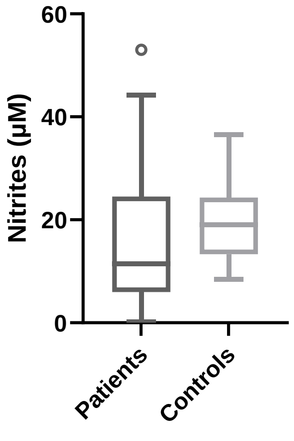

(a)

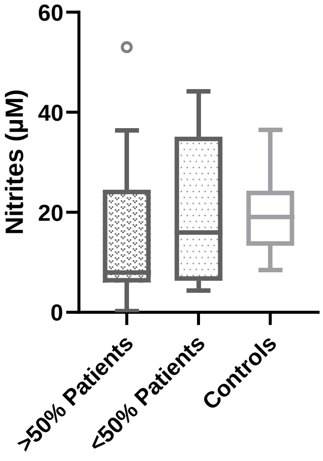

(b)

Supplementary Figure S1. Nitrite levels in serum. Nitrate/nitrite levels in serum were evaluated by the Griess method. **(a)** Patients (n=23) and Controls (n=23) and **(b)** dividing by the degree of stenosis (n=15 ≥50% and n=8 <50%).

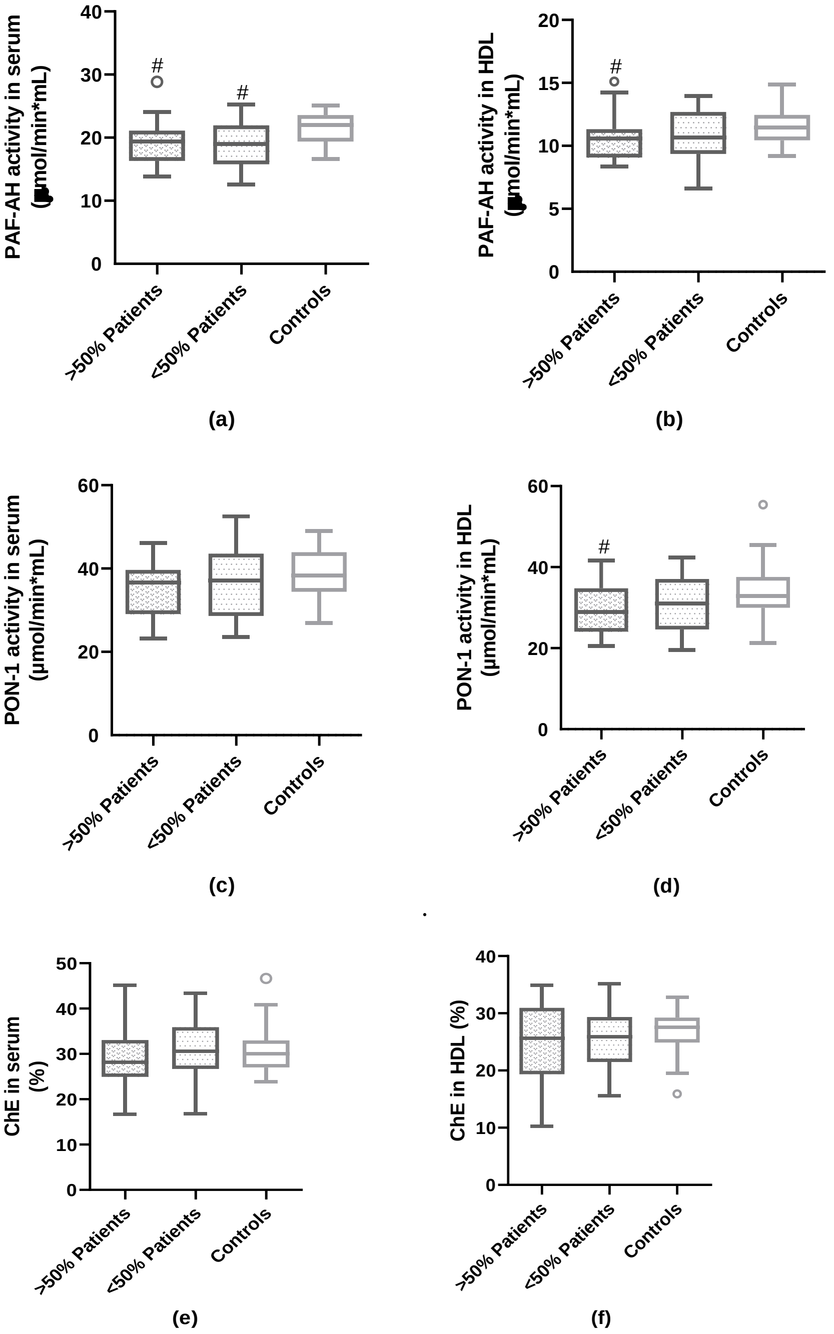

Supplementary Figure S2. Parameters of HDL functionality. PAF-AH and PON-1 activities and ChE were analyzed in apoB-depleted serum from Controls and patients divided by the degree of stenosis. PAF-AH and PON-1 activities were calculated from the slope of the enzymatic activity **(a)** and **(b)**, respectively; and ChE capacity was analyzed in THP1-CD14 macrophages after 24 hours' incubation **(c)**. Patients (n=46, of whom n= 26 ≥50% stenosis degree); Controls (n=26); # vs Controls, p≤0.05

Supplementary Table S1. Clinical characteristics of the patients

	<50% group (n=27)	≥50% group (n=37)	p	All stroke patients (n=64)	Control group (n=27)	p
Age, mean (SD)	73.6 (9.7)	75.9 (9.7)	0.334	74.9 (9.7)	73.8 (5.7)	0.564
Sex (female), n (%)	5 (18.5)	11 (29.7)	0.306	16 (25)	6 (22.2)	0.283
BMI, mean (SD) (kg/m^2)	25.85 (2.59)	25.35 (3.88)	0.562	25.56 (3.38)	27.37 (3.87)	**0.028**
Regular exercise (PACE ≥4), n (%)	13 (48.2)	17 (46.0)	0.862	30 (46.9)	20 (74.1)	**0.017**
PREDIMED score, md (IQR)	9 (5-10)	9 (7-9)	0.751	9 (6-10)	8 (7-9)	0.098
Current Smoking, n (%)	4 (14.8)	10 (27.0)	0.243	14 (21.9)	0 (0.0)	**0.008**
Hypertension, n (%)	26 (96.3)	27 (73.0)	**0.015**	53 (82.8)	18 (66.7)	0.089
Diabetes, n (%)	9 (33.3)	18 (48.7)	0.221	27 (42.2)	5 (18.5)	**0.031**
Dyslipidemia, n (%)	20 (74.1)	23 (62.2)	0.316	43 (67.19)	7 (25.9)	<0.001
Active or recent cancer (<5years), n (%)	2 (7.4)	3 (8.1)	0.918	5 (7.8)	2 (7.4)	0.947
Coronary artery disease, n (%)	7 (25.9)	10 (27.0)	0.922	17 (26.6)	-	-

Prior stroke, n (%)	5 (18.5)	6 (16.2)	**<0.001**	11 (17.2)	-	-
Prior antiplatelet therapy, n (%)	15 (55.6)	22 (59.5)	0.755	37 (57.8)	5 (18.5)	**<0.001**
Prior statin therapy, n (%)	15 (55.6)	20 (54.1)	0.905	35 (54.7)	6 (22.2)	**0.005**
Baseline NIHSS, md (IQR)	3 (1-6)	2 (0-3)	0.324	2 (0-5)	-	-
Intravenous fibrinolysis, n (%)	7 (25.9)	4 (10.8)	0.113	11 (17.2)	-	-
Acute lesion on neuroimaging, n (%)	20 (74.1)	26 (70.3)	0.738	46 (71.9)	-	-
Stroke etiology, n (%)						
- Atherothrombotic	0 (0.0)	31 (83.8)		31 (83.8)		
- Lacunar	7 (25.9)	0 (0.0)		7 (25.9)		
- Cryptogenic	20 (74.1)	0 (0.0)	**<0.001**	20 (74.1)	-	-
- Undetermined (two causes)	0 (0.0)	6 (16.2)		6 (16.2)		

BMI (body mass index); PACE (physician-based assessment and counseling for exercise); NHISS (National Institutes of Health Stroke Scale).

Student's t-test or the Wilcoxon rank-sum test (when a non-parametric test was required) for continuous variables, and the $\chi 2$ test for categorical variables were used to compare groups; $p<0.05$ indicates significant differences.

Supplementary Table S2. Serum MDA levels and antioxidant capacity

	Lipid oxidation		Antioxidant capacity (DPPH)	
	MDA (μM)	n	Decrease Abs 520 nm (%)	n
Controls	6.15 (4.58-8.91)	10	52.03 (33.31-65.88)	10
<50% degree of stenosis	5.75 (3.40-7.77)	21	47.16 (33.24-68.24)	10
≥50% degree of stenosis	5.10 (3.18-8.43)	18	46.49 (28.11-64.59)	14

MDA (malondyaldehide); DPPH (2,2-diphenyl-1-picrylhydrazyl); md (IQR).

Supplementary Table S3. Correlation between the proportion of LDL(-) and oxLDL with other lipid-related parameters

	LDL(-)		oxLDL	
	Spearman's rho	p	Spearman's rho	p
Lipids and apolipoproteins				
Triglycerides	-0.17	0.190	0.13	0.270
Total cholesterol	-0.05	0.686	-0.21	0.076
VLDLc	-0.17	0.195	0.13	0.270
LDLc	0.08	0.476	-0.20	0.083
HDLc	0.18	0.157	-0.19	0.105
LDLc/HDLc ratio	-0.20	0.125	-0.05	0.669
NEFA	-0.21	0.110	-0.01	0.922
apoB	-0.09	0.466	-0.19	0.105
apoA-I	-0.05	0.659	-0.22	0.065
apoA-II	-0.12	0.353	-0.16	0.171
apoE	-0.12	0.256	-0.16	0.180
apoC-III	-0.15	0.233	-0.29	**0.014**
apoJ	0.12	0.269	0.34	**0.003**
apoJ/PON-1 activity ratio	0.08	0.507	0.25	**0.035**
oxLDL/LDLc	0.14	0.226	-	-
Total PAF-AH activity	-0.08	0.593	-0.42	**<0.001**
Parameters of HDL functionality				
PAF-AH activity	0.02	0.901	-0.35	**0.002**
PON-1 activity	0.14	0.342	0.04	0.745
ChE	0.17	0.228	0.04	0.743

Plasma inflammatory markers				
sICAM-1	-0.14	0.310	0.06	0.625
sVCAM-1	-0.15	0.273	0.04	0.733
FKN	-0.11	0.413	0.14	0.255

VLDLc (very low-density lipoprotein cholesterol); LDLc (low-density lipoprotein cholesterol); HDLc (high-density lipoprotein cholesterol); NEFA (non-esterified fatty acid); apo (apolipoprotein); PON-1 (paraxonase-1); oxLDL (oxidized LDL); PAF-AH (platelet-activating factor acetylhydrolases); ChE (cholesterol efflux); sICAM-1 (soluble intercellular adhesion molecule 1); sVCAM-1 (soluble vascular adhesion molecule 1); FKN (fractalkine).

Spearman's correlation was used to analyze the correlation between LDL(-) and other lipid-related parameters; n = 91 (includes patients and controls); $p<0.05$ indicates significant differences.

Supplementary Table S4. Clinical variables and lipid parameters in patients with predominantly hypoechoic or hyperechoic plaque

	Predominantly hypoechoic (n=19)	Predominantly hyperechoic (n=45)	*p*
Clinical characteristics			
Age, mean (SD)	73.9 (10.5)	75.4 (9.4)	0.568
Sex (female), n (%)	5 (26.3)	11 (24.4)	0.874
BMI, median (IQR)	25.7 (23.8-26.7)	25.9 (23.3-27.7)	0.786
Regular exercise (PACE ≥4), n (%)	10 (52.6)	20 (44.4)	0.549
PREDIMED score, md (IQR)	8 (6-9)	9 (6-10)	0.284
Current Smoking, n (%)	3 (15.8)	11 (24.4)	0.444
Hypertension, n (%)	15 (79.0)	38 (84.4)	0.594
Diabetes, n (%)	6 (31.6)	21 (46.7)	0.264
Dyslipidemia, n (%)	7 (36.8)	36 (80.0)	**0.001**
Coronary artery disease, n (%)	4 (21.1)	13 (28.9)	0.517
Prior stroke, n (%)	2 (10.5)	9 (20.0)	0.359
Prior antiplatelet therapy, n (%)	9 (47.4)	28 (62.2)	0.272
Prior statin therapy, n (%)	6 (31.6)	29 (64.4)	**0.016**
Lipids and apolipoproteins			
Triglycerides (mM), md (IQR)	1.37 (0.96-1.73)	1.16 (0.92-1.47)	0.340
Total cholesterol (mM), m± sd/md (IQR)	4.37 (3.04-4.81)	3.72 (2.87-4.44)	0.366
VLDLc (mM), md (IQR)	0.27 (0.19-0.35)	0.23 (0.18-0.29)	0.366
LDLc (mM), md (IQR)	3.02 (1.85-3.31)	2.33 (1.76-2.90)	0.228
HDLc (mM), m± sd/md (IQR)	1.03 (0.80-1.19)	1.03 (0.76-1.33)	0.797

LDLc/HDLc ratio, md (IQR)	2.64 (2.08-3.21)	2.42 (1.83-2.93)	0.130
NEFA (mM), md (IQR)	0.30 (0.22-0.39)	0.47 (0.30-0.69)	**0.011**
apoB (g/L), md (IQR)	0.68 (0.57-0.83)	0.68 (0.58-0.81)	0.797
apoA-I (g/L), md (IQR)	1.21 (1.08-1.27)	1.27 (1.12-1.48)	0.201
apoA-II (g/L), mean (SD)	0.28 (0.1)	0.29 (0.1)	0.696
apoE (g/L), mean (SD)	0.03 (0.0)	0.04 (0.0)	0.122
apoC-III (g/L), md (IQR)	0.02 (0.01-0.11)	0.06 (0.03-0.09)	0.134
apoJ (mg/L), md (IQR)	180.6 (142.1-217.2)	175.9 (151.7-218.3)	0.721
LDL(-) (%), md (IQR)	9.5 (7.1-11.7)	7.4 (5.6-9.9)	**0.010**
oxLDL (U/mmol LDLc), md (IQR)	15.2 (5.37-18.2)	11.9 (8.16-17.01)	0.709
PAF-AH activity (μmol/min*mL), mean (SD)	20.3 (3.7)	18.6 (3.2)	0.156
Parameters of HDL functionality			
PAF-AH activity (μmol/min*mL), md (IQR)	56.2 (48.2-59.3)	55.7 (53.7-59.2)	0.463
PON-1 activity (μmol/min*mL) , md (IQR)	28.5 (25.4-32.3)	30.2 (24.2-37.5)	0.422
ChE (%), md (IQR)	27.8 (19.4-30.5)	24.1 (21.1-28.9)	0.453

BMI (body mass index); PACE (physician-based assessment and counseling for exercise); VLDLc (very low-density lipoprotein cholesterol); LDLc (low-density lipoprotein cholesterol); HDLc (high-density lipoprotein cholesterol); NEFA (non-esterified fatty acid); apo (apolipoprotein);LDL(-) (electronegative LDL); oxLDL (oxidized LDL); PAF-AH (platelet-activating factor acetylhydrolases); PON-1 (paraxonase-1); ChE (cholesterol efflux).

Student's t-test or the Wilcoxon rank-sum test (when a non-parametric test was required) for continuous variables, and the $\chi 2$ test for categorical variables were used to compare groups; $p<0.05$ indicates significant differences.

Supplementary Table S5. Clinical variables and lipid parameters in patients with diffuse or non-diffuse intraplaque neovascularization

	Diffuse intraplaque neovascularization (n=14)	Non-diffuse neovascularization (n=24)	*p*
Clinical characteristics			
Age, mean (SD)	75.1 (10.2)	75.3 (9.6)	0.937
Sex (female), n (%)	6 (42.9)	5 (20.8)	0.149
BMI, md (IQR)	26.2 (23.3-29.0)	26.2 (25.6-27.1)	0.364
Regular exercise (PACE ≥4), n (%)	8 (6-9)	8 (8-10)	0.501
PREDIMED score, md (IQR)	8 (6-9)	9 (6-10)	0.521
Current Smoking, n (%)	3 (21.4)	5 (20.8)	0.965
Hypertension, n (%)	11 (78.6)	19 (79.2)	0.965
Diabetes, n (%)	6 (42.9)	11 (45.8)	0.859
Dyslipidemia, n (%)	7 (50.0)	18 (75.0)	0.117
Coronary artery disease, n (%)	1 (7.1)	6 (25.0)	0.171
Prior stroke, n (%)	2 (14.3)	4 (16.7)	0.846
Prior antiplatelet therapy, n (%)	5 (35.7)	15 (62.5)	0.111
Prior statin therapy, n (%)	6 (42.9)	14 (58.3)	0.357
Lipids and apolipoproteins			
Triglycerides (mM), md (IQR)	1.38 (0.87-1.87)	1.36 (1.09-1.79)	0.650
Total cholesterol (mM), md (IQR)	4.24 (3.54-4.81)	3.67 (3.05-4.74)	0.380
VLDLc (mM), md (IQR)	0.27 (0.17-0.37)	0.27 (0.22-0.36)	0.672
LDLc (mM), md (IQR)	3.03 (1.92-3.26)	2.40 (1.90-3.31)	0.468
HDLc (mM), md (IQR)	1.08 (0.91-1.27)	0.95 (0.75-1.18)	0.263
LDLc/HDLc ratio, md (IQR)	2.57 (1.83-3.34)	2.53 (2.15-3.06)	0.976

NEFA (mM), md (IQR)	0.37 (0.19-0.44)	0.38 (0.28-0.55)	0.431
apoB (g/L), md (IQR)	0.72 (0.61-0.96)	0.62 (0.57-0.82)	0.122
apoA-I (g/L), md (IQR)	1.41 (1.15-1.51)	1.18 (1.08-1.34)	0.067
apoA-II (g/L), mean (SD)	0.30 (0.1)	0.28 (0.1)	0.451
apoE (g/L), mean (SD)	0.04 (0.0)	0.04 (0.0)	0.525
apoC-III (g/L), md (IQR)	0.08 (0.03-0.12)	0.04 (0.01-0.08)	0.164
apoJ (mg/L), md (IQR)	163.2 (147.8-201.3)	167.0 (140.5-202.1)	0.802
LDL(-), md% (IQR)	9.0 (7.1-11.7)	7.0 (4.6-9.2)	**0.033**
oxLDL (U/mmol cLDL), md (IQR)	15.5 (6.2-17.1)	11.9 (8.8-14.5)	0.569
PAF-AH activity (μmol/min*mL), mean (SD)	20.0 (4.1)	18.9 (3.2)	0.402
Parameters of HDL functionality			
PAF-AH activity (μmol/min*mL), md (IQR)	10.7 (10.2-11.08)	11.1 (10.1-11.7)	0.357
PON-1 activity (μmol/min*mL), md (IQR)	29.7 (26.8-32.8)	27.2 (23.0-30.8)	0.313
ChE (%), md (IQR)	19.4 (17.5-20.5)	18.3 (16.9-21.5)	0.929

BMI (body mass index); PACE (physician-based assessment and counseling for exercise); VLDLc (very low-density lipoprotein cholesterol); LDLc (low-density lipoprotein cholesterol); HDLc (high-density lipoprotein cholesterol); NEFA (non-esterified fatty acid); apo (apolipoprotein); LDL(-) (electronegative LDL); oxLDL (oxidized LDL); PAF-AH (platelet-activating factor acetylhydrolases); PON-1 (paraxonase-1); ChE (cholesterol efflux).

Student's t-test or the Wilcoxon rank-sum test (when a non-parametric test was required) for continuous variables, and the $\chi 2$ test for categorical variables were used to compare groups; $p<0.05$ indicates significant differences.
